# The acceptability of intermittent screening and treatment versus intermittent preventive treatment during pregnancy: results from a qualitative study in Northern Ghana

**DOI:** 10.1186/1475-2875-13-432

**Published:** 2014-11-18

**Authors:** Christopher Pell, Arantza Meñaca, Samuel Chatio, Abraham Hodgson, Harry Tagbor, Robert Pool

**Affiliations:** Centre for Social Science and Global Health, University of Amsterdam, Amsterdam, The Netherlands; Centre de Recerca en Salut Internacional de Barcelona (CRESIB), Hospital Clínic-Universitat de Barcelona), Barcelona, Spain; Departamento de Antropología Social, Universidad Complutense de Madrid, Madrid, Spain; Navrongo Health Research Centre, Navrongo, Ghana; Research and Development Division, Ghana Health Service, Accra, Ghana; Department of Community Health, School of Medical Sciences, Kwame Nkrumah University of Science and Technology, Kumasi, Ghana

**Keywords:** IPTp, Intermittent screening and treatment (IST), Malaria, Pregnancy, Acceptability

## Abstract

**Background:**

Affecting mother and child, malaria during pregnancy (MiP) provokes a double morbidity and mortality burden. Within a package of interventions to prevent MiP in endemic areas, the WHO currently recommends intermittent preventive treatment (IPTp). Concerns about anti-malarial resistance have however prompted interest in intermittent screening and treating (IST) as an alternative approach to IPTp. IST involves screening for malaria infection at scheduled antenatal care (ANC) clinic visits and treating malaria cases. In light of the need to comprehensively evaluate new interventions prior to roll out, this article explores the acceptability of IST with artemether-lumefantrine (AL) compared to IPTp with sulphadoxine-pyrimethamine (SP) and in Upper East Region, northern Ghana.

**Methods:**

Data were collected alongside an open-label, randomized, controlled trial of IST-AL and IPTp-SP in Kassena-Nankana District. Thirty pregnant women enrolled in the clinical trial participated in six focus group discussions. Ten in-depth interviews were carried out with clinical trial staff. Observations were also made at the health facilities where the clinical trial took place.

**Results:**

Trial participants were generally willing to endure the discomfort of the finger prick necessary for a rapid diagnostic test for malaria and this reflected a wider demand for diagnostic techniques. Reports of side effects were however linked to both trial anti-malarials. Direct complaints about SP were particularly severe with regard to women’s experience of vomiting. Although the follow-up treatment doses of AL for IST were not supervised, based on blister inspection and questioning trial, staff were confident about participants’ adherence to the treatment course. One case of partial adherence to the AL treatment course was reported.

**Conclusion:**

Despite the discomfort of the finger prick required to perform the intermittent malaria screening, trial participants generally expressed more positive sentiments towards IST-AL than IPTp-SP. Nonetheless, questions remain about adherence to a multiple dose anti-malarial regimen during pregnancy, particularly in endemic areas where MiP is often non-symptomatic. Any implementation of IST must be accompanied by appropriate health messages on adherence and the necessary training for health staff regarding case management.

## Background

Malaria during pregnancy (MiP) is a major global health concern [1, 2]. It provokes a double burden of morbidity and mortality because it affects both mother and child, particularly in endemic regions of sub-Saharan Africa [1]. It compounds or provokes maternal anaemia, which, when severe, increases the risk of maternal death and it has been estimated that, globally, malaria-related anaemia causes around 10,000 maternal deaths each year [3]. It can also result in low birth weight (estimated to cause around 100,000 infant deaths in Africa [3]), preterm delivery, congenital infection, and reproductive loss [1].

In response, the WHO recommends several interventions for MiP prevention and control in areas of stable, moderate to high malaria transmission: sleeping under insecticide-treated bed nets (ITNs) during pregnancy (preferably long-lasting insecticide-treated nets (LLINs) [4]; case management using appropriate anti-malarial regimens [5]; and intermittent preventive treatment (IPTp) [6]. IPTp involves the administration, as early as possible in the second trimester, of treatment doses of an anti-malarial as part of routine antenatal care (ANC). Although increasing resistance poses questions about its continued efficacy [7], sulphadoxine-pyrimethamine (SP) is currently used for IPTp in many sub-Saharan African countries [8].

Coverage of IPTp-SP varies across sub-Saharan Africa and part of this has been attributed to operational problems, including stock-outs and confusion about timing of doses [9]. Concerns about the implications of IPTp-SP for the prevalence of anti-malarial resistance and its continued effectiveness [7, 10] have also prompted interest in a more targeted approach. Intermittent screening and treating (IST), which involves screening women for malaria infection using a rapid diagnostic test (RDT) at scheduled ANC clinic visits and treatment of those who are positive with an effective anti-malarial, has been proposed as an alternative strategy for MiP prevention [11].

To date, the effectiveness of IST during pregnancy has undergone relatively limited evaluation. The results from a randomized, controlled, non-inferiority trial of IPTp with SP *versus* IST with SP or amodiaquine + artesunate (AQ + AS) in central Ghana suggested that this was an effective and safe strategy for the prevention of MiP [11]. Furthermore, a qualitative study linked to this trial illustrated that IST was hardly noticed as an addition to the package of ANC services provided [12]. These studies however stressed the need to strengthen the evidence base for IST through its comprehensive evaluation in different malaria transmission and social contexts.

Research evaluating IPT-SP has included qualitative studies of its acceptability across several African sites. These studies have highlighted that in a variety of settings SP was viewed as harmful during pregnancy [13–15]. However, in some contexts, in spite of concerns about SP, women took the IPTp-SP because of the confidence that they placed in those delivering it [16, 17]. Moreover, a multi-site study of the social and cultural context of MiP interventions highlighted how women generally attended ANC and, apart from a few exceptions, adhered to IPTp-SP as part of a general acceptance of the package of ANC intervention, even though they disliked the side effects that they associated with it (often vomiting) [18]. However, the addition of the finger-prick and RDT means that this begrudging acceptance as part of ANC cannot be extrapolated to the possible implementation of IST. With the possibility of a future roll-out, it is necessary to examine how recipients of IST react to this intervention – in terms of their attitudes towards it and their behaviours – particularly with regard to the addition of a finger-prick, RDT and the curative anti-malarial regimen to ANC. Examining this response is not only relevant for MiP prevention and control but also potentially for ANC attendance and therefore mother and child health in general.

Under the auspices of the MiP Consortium [19] and as part of a wider programme of research on the social and cultural context of MiP [20–22], this article addresses the question of whether IST is acceptable in a context that is environmentally, socially, culturally and ethnically [23] distinct from the setting of previous research on IST in central Ghana [12]. Indeed, previous research highlighting differences in attitudes and behaviours regarding malaria prevention and control between pregnant women in central and northeastern Ghana [18] emphasizes the need conduct further research on the acceptability of IST in the latter area. Also, the seasonal nature of malaria transmission in this area makes it a particularly relevant context for research on the acceptability of IST; questions about the continued effectiveness of IPTp-SP and discussions of alternative strategies tend to focus on such malaria transmission settings [24].

Therefore, to explore the acceptability of IST compared to IPTp-SP in Upper East Region, northeastern Ghana, this study describes and analyses the attitudes and behaviours of pregnant women related to these interventions when delivered as part of a clinical trial. Conducting this research in the context of a clinical trial led to a focus on the potential recipients of IST rather than those delivering the intervention. Although this limits the scope of the study, it is an opportunity to identify issues that can be addressed prior to implementation.

## Methods

### Settings

Data were collected alongside an open-label, randomized, controlled trial of IST and IPTp. The clinical trial involved recruitment of over 5,000 primigravidae and secundigravidae during ANC visits. As part of their participation in the study all the women were provided with LLINs and required to use them regularly. Women in the IST arm were screened for malaria by trained staff using RDT and those with a positive result were treated with artemether-lumefantrine (AL), following a three-day regimen of two times four tablets of 20 mg artemether/120 mg lumefantrine. The first dose was administered at the health facility under supervision, whereas the remaining doses were self-administered by the participants. SP-IPT involves ingestion of SP (1,500 mg sulphadoxine/75 mg pyrimethamine) directly observed by ANC staff. As part of the clinical trial, participants were encouraged to give birth in a health facility under the supervision of trial staff and, after birth, each participant’s placenta was collected for further clinical analysis. For all trial participants, after birth, maternal haemoglobin (via finger prick) and birth weight were recorded and, six weeks, post-partum, women were visited at home and a blood sample was taken (finger prick).

The clinical trial and data collection for the acceptability study took place Kassena-Nankana Districts, Upper East Region, northeast Ghana. This area is part of the Sahel and experiences an annual rainy season during which people grow millet, maize, and vegetables for subsistence. During the rest of the year, part of the population migrates to other regions. The *Kassena* and the *Nankani*, make up almost 90% of the population of the district [25]. Here, malaria transmission is perennial but there is a seasonal pattern with a transmission peak that coincides with the major rains (May to October) and the low rates of infection during the dry season [26].

### Data collection

Data on the acceptability of IST and IPTp were collected during November and December 2010, the last two months of a year-long period of fieldwork for broader research on the social and cultural context of MiP [18, 20–22]. During these two months, together with several field assistants who had been extensively trained as part of the broader research, the second author (AM) conducted six focus group discussions (FGDs) with 30 pregnant women participating in the clinical trial (see Table 1 for details); ten in-depth interviews (IDIs) with staff working on the clinical trial (midwifes, trial coordinator, field managers, and field staff), and made observations at the health facilities where the clinical trial was taking place.

**Table 1:** **Summary of FGD participants**

FGD location	Trial arm	Participant age	Participant no. of children	Participant education level
Kassena-Nankana East Health Centre	IPTp	20	1	Primary one
		Unknown	1	None
		25	0	Primary four
		22	0	Senior secondary
		20	1	None
		22	2	Junior secondary
Yua CHPS	IST	28	1	Senior secondary
		20	0	Junior secondary
		18	0	Junior secondary
		20	0	None
		Unknown	0	None
Paga Health Centre	IPTp and IST	20	2	Primary
		19	1	None
		19	0	Senior secondary
		25	1	Primary
Sirigu Clinic	IPTp and IST	22	0	Junior secondary
		22	0	Primary
		26	1	Primary
		26	0	Senior secondary
		20	0	Junior secondary
Central Clinic	IPTp	20	1	Primary four
		22	1	Junior secondary
		24	1	Primary five
		20	1	Junior secondary
		23	1	Junior secondary
Central Clinic	IPTp and IST	22	1	Junior secondary
		24	1	Senior secondary
		21	1	None
		22	1	Junior secondary
		24	2	Primary six

Trial participants were selected to ensure that women from both arms were interviewed from a range of health facilities (including public health centres, private clinics and Community-based Health Planning and Service [CHPS] posts) where the trial was conducted. The FGDs included women from a single or both trial arms and were undertaken in an attempt to incorporate as many trial participants as possible in the limited time frame for fieldwork and to elicit normative responses as well as individual experiences. Trial staff with varied roles and responsibilities were selected for interview to ensure as diverse as possible range of experiences and opinions were included for analysis. Because trial staff were selected from different levels of the hierarchy, individual interviews were deemed most relevant to ensure that the responses of lower level staff were not influenced by the presence of superiors. Across four health facilities, observations were made of with recruitment and the daily activities of the clinical trial, which included the finger-prick for IST (an element of IST that has potential ramifications for its acceptability).

FGDs and IDIs were conducted using topic guides that focused on respondents’ experiences of the trial procedures, particularly the finger pricks used as part of malaria diagnosis with an RDT and the medication delivered as part of the trial. In keeping with the anthropological approach of the broader study, IDIs and FGDs were conducted in a flexible, reflexive and iterative manner that adapted to the topics emerging during data collection. The interviews were conducted in *Kassem*, *Nankani* or English and translated into English (if necessary) for analysis. To maintain data quality and check for errors, trained field staff conducted transcription and translation under the supervision of the field coordinator (SC) and AM. Both SC and AM also read the transcripts shortly after their completion and discussed their content with the translator/transcriber.

The resulting interview and focus group transcripts, along with the field notes from observations, were analysed together using QSR NVivo 10. A codebook was developed using largely pre-established categories based on the original research question but also modified as themes emerged from the data. CP undertook coding and analysis in consultation with AM.

### Ethics statement

Ethical clearance was obtained from the Clinical Research Ethics Committee, Hospital Clinic-University of Barcelona. Additional local ethical clearance was obtained in Ghana from the Institutional Review Board of the Navrongo Health Research Centre, Navrongo. As approved by the ethics review committees, informed consent was obtained orally from study participants. With the permission of respondent(s), interview/FGD was audio recorded (along with the informed consent itself).

## Results

From the focus groups and interviews with trial participants and staff, several key issues were identified as particularly relevant to the acceptability of IST-AL and IPTp-SP. These included attitudes and behaviour relating to malaria screening with an RDT along with the perceived side effects, efficacy and adherence of both trial drugs. The clinical trial context also influenced how these interventions were received.

### Screening for malaria

The RDT used to diagnose malaria amongst trial participants entailed blood samples being drawn via finger prick. Interviewed trial participants viewed this as a painful procedure but many expressed a willingness to endure the discomfort and discover whether they were suffering malaria.

Respondent (R) 2: *For me, I think the prick on your hand to test is far better. When you are tested they will know whether you have serious malaria or not. If you have the serious one, they will know what to do.*

R3: *For me, I think they will have to test before the give the medicine. If they don’t test how can they start to give you medicine without testing you to know what the problem is*.

FGD, women in IPTp and IST arms (Central Clinic)

However, for some, the prospect of a finger prick provoked sufficient fear as to prefer foregoing diagnosis and receiving an anti-malarial regardless of a confirmed infection.

*I also prefer to take the [SP] because at times when they are going to prick your hand they won’t warn you, which makes it very painful. That is why I just don’t like it.*

FGD participant, women in IPTp and IST arms (Paga Health Centre)

Health staff reported that most women were unhappy with the finger-prick. Observations also suggested that women found the process uncomfortable, often averting their eyes during the procedure. Furthermore, the trial field coordinator described how some women would be deterred from participating in the trial because of the finger pricks.

*Some of [the women] say that they are fed up. You know, when they come to us, we do pricking and take blood samples, to know their HB, if they have malaria and so many other things. Some of the women fear pricking and so, when we invite them [to participate in the trial] they won’t come.*

IDI, trial field coordinator

The concerns were particularly related to the pain and discomfort of the finger-prick but there had also been complaints about a lack of compensation for the follow-up blood testing of participants from both trial arms. One field worker described how trial participants’ criticisms had led to trial staff providing a bar of soap as an incentive after each finger prick conducted at all trial participants’ homes six weeks post partum. Although trial staff were also aware of problems with blood sampling at other research sites, particularly with regard to rumours of ‘blood stealing’, they had not encountered such rumours, which they linked to the local population’s familiarity with medical research, the small quantity of blood that was sampled and the explanations that were given during the consent process about the reasons for blood sampling. Similarly, there was little reported resistance to the *post-partum* collection and sampling of the participants’ placentas: although potentially divisive, women were placated by the explanations that they received on enrolment. With regard to clinical research more generally in this research site, one trial staff member explained:

Respondent (R): *When we are taking the blood we tell them what we are going to use the samples for. We also tell them that we are not taking much of their blood. So at the end they know that we are using their blood to know their HB and to see if they have malaria but not to give it to some other people. So they cooperate with us.*

Interviewer (I): *So you think that if you were taking more blood there would be more problems?*

R: *Yes. If we were taking a lot of blood they may think that we are selling it for money or that we are donating it to some other people.*

I: *Has it ever happened like that in any trial here in Navrongo?*

R: *No. In the projects that I know they also don’t take much blood.*

IDI, trial field coordinator

Indeed, the observations of the consent process suggest that the trial staff were careful to spell out that trial participation entailed malaria diagnosis via finger-prick and use of the placenta for research. Women were also given explanations as to why these procedures were conducted.

### Trial drugs: side effects, perceived efficacy and adherence

#### Artemether-lumefantrine (AL)

Women’s reports regarding the efficacy of AL were mixed. As the excerpt below indicates, the trial participants experience of the ‘yellow malaria drugs’ had varying degrees of success in terms of treating bouts of malaria. Indeed, respondent 1 in the same discussion explained this in terms of everyone having a ‘different body’. There was however a general absence of direct references to side effects caused by AL.

I: *So, this malaria that we are talking about, did it end with the drugs [AL] and come again or did it never even with the drugs?*

R3: *For me, when I was taking the drug the malaria was still with me.*

R1: *For me, when I finished taking the drugs the malaria also stopped for some time before it came back.*

R5: *In my case, when I took the drug I was ok. I did not have malaria again.*

FGD, IST-arm participants (Yua CHPS)

In contrast, trial staff described trial participants complaining about AL causing side effects, including nausea, dizziness and appetite suppression. Complaints also centered on the perceived high number of tablets that a treatment course of AL entailed (a total of 24 tablets over three days). One woman was reportedly dissuaded from taking AL by another health worker (who had no association with the trial) who advised that AL was too ‘strong’ for use during pregnancy and that it could lead to miscarriage.

R: *[Trial participants] are complaining about the Coartem [AL].*

I: *What are some of the complaints that they talk about?*

R: *There are many. They say that to take four [tablets per dose] is too much. They are complaining about the number. They say that even taking one is not easy for them. Some of them don’t eat well before they come [to the health facility], so when they take the medicines they feel like vomiting.*

IDI, trial midwife

In spite of the concerns, trial staff were confident about participants’ non-observed adherence to the AL treatment course. Although the women took all but the first dose of AL unobserved, on follow-up visits trial staff collected the empty AL blisters. They based their assertions about adherence on this and also on women’s positive reports of the efficacy of AL in terms of reducing their symptoms of malaria. There was however one report of non-adherence caused by AL-associated side effects: the trial field supervisor had visited a woman whose experiences of dizziness had led her to stop taking the tablets. Based on his advice about the malaria infection not being cured, the participant restarted the AL course. Another woman described how she had taken the treatment course over four days rather than three.

I: *So for the Coartem [AL] they take all even if they complain that they cannot eat well?*

R: *Yes, though they complain they still take it.*

IDI, trial coordinator

#### Sulphadoxine-pyrimethamine (SP)

Trial participants complained directly of side effects associated with SP. These included vomiting, nausea, diarrhoea, appetite suppression or increase, general body weakness, and dizziness. Based on their negative experiences, some women questioned trial staff about the benefits of such drugs, and were told that they prevented malaria and were beneficial for both women and their children. The side effects also varied across the IPTp-SP doses and women did not necessarily experience side effects with every dose.

R1: *It is the white large medicines [SP] that are very difficult to take. When you take them you vomit all the time and you also have body weakness and you lose appetite.*

[…]

R3: *Yes, those three tablets [of SP] are really a problem. When you take them and you get to the house it is always like you are drunk…*

R2: *When I take them I become very weak and eat a lot…I have never vomited after taking them.*

I: *Is there any of you here that the [SP] has no effect on?*

R4: *The first and second time that I took the big white medicine, I did not have any problem but the third time that I took it I vomited lots.*

R5: *When I took it for the first time and got to the house it was like I had diarrhoea; the second time, the same thing happened but with body weakness; and the third time, when I got to the house after taking the medicine I was a little dizzy but it was just for some short time.*

I: *And has anybody taken it and had no problem?*

R6: *I also had a problem when I took them it made feel like vomiting but I could not vomit.*

FGD, IPTp-arm participants (Kassena-Nankana East Health Centre)

One trial staff member explained the frequency of SP-related side effects in terms of the poverty and resultant poor diets of pregnant women in the area.

*You know, in this part of the country the poverty is high. Some people do not eat until afternoon or so. So, when pregnant women take the SP without food they become dizzy. So we always tell them at least to take [flour and water] with groundnuts before they take the drugs.*

IDI, trial midwife

In light of the side effects, trial staff suggested that if SP was not delivered under directly observed therapy (DOT) conditions, women would discard the tablets. However, the staff and even one trial participant recognized the effectiveness of IPTp with SP in terms of reducing the negative effects of MiP, particularly a reduction in miscarriage and improvement in birth weight.

*I think the white [SP] tablets are very good because the drug prevents the child from getting malaria. Now you can see that when you give birth to your children they look very strong. Before, many people were losing their pregnancy because of malaria. But now there is prevention.*

FGD, participants from both trial arms (Sirigu CHPS)

### IPTp and IST within a clinical trial

Trial participants’ attitudes towards the interventions were inevitably influenced by the context in which they were delivered. Participation in the clinical trial was generally popular, particularly because of the health care and other benefits that were offered. Indeed, although the trial enrolment criteria restricted participation to women of parity two and below, health staff reported that women of higher parity sought to enrol in the study, citing the benefits, particularly an ITN (which were not always provided free of charge at ANC) and requested that staff ignored this exclusion criterion. The local research institute, in collaboration with various overseas partners, has conducted a range of clinical studies in the area and trial participants generally valued these activities, again, particularly for the benefits that participants received, which reportedly included lifts to and from health facilities and food. Previous experiences of such benefits, and anticipation that these would be provided, also prompted women to participate in this particular study. Benefits were however the same for participants in both arms of the trial. Therefore they are likely to have affected attitudes towards both interventions in a similar manner.

## Discussion

Several issues were particularly relevant to the acceptability of IST with AL and IPTp with SP in the context of a clinical trial in Upper East, Ghana. These include the response to the finger-prick blood sampling required to perform malaria screening using an RDT and the attitudes towards the anti-malarials, in terms of their ease of use, perceived efficacy and side effects. Although the data were collected in the context of a clinical trial, the findings provide useful pointers for the possible future implementation of IST (presumably replacing IPTp) as part of standard ANC procedures. To offer some insights for the potential implementation of IST, these findings are discussed below with reference to the broader study on the social and cultural context of MiP and other research of which these data form a small part [18, 20–22] and other relevant research.

### Screening

Trial participants valued the opportunity to receive a malaria diagnosis with an RDT and this reflected a broader appreciation of malaria (and other) diagnostic technologies. In this regard, IST is potentially more appealing to women than IPTp. Furthermore, in general, pregnant women interviewed for the wider study on the social and cultural context of MiP at this and other sites [18] viewed diagnosis with an RDT (or laboratory test) positively. Positive attitudes towards RDTs (albeit for other populations groups) have also been highlighted in southern and central Ghana [27, 28]: in both these studies, RDTs were viewed as an indication of quality of care.

The value placed on malaria diagnostic tests must however be viewed within the complex relationship between biomedically defined malaria, local illness concepts that equate roughly with malaria and the ‘normal’ symptoms of pregnancy as previously described in Ghana and elsewhere [22]. This relationship often entails a *partial* overlap in terms of symptoms. Therefore a bout of ‘malaria’ (or the roughly equivalent local illness), which is labelled according to an individual’s symptoms, may not be diagnosed as biomedically defined malaria. Indeed, for respondents in Upper East (and elsewhere), a positive malaria test result was viewed as confirming an infection. However, a negative result was sometimes questioned and this potentially leads to doubts about the accuracy of malaria tests.

A recent systematic review has highlighted a paucity of research on RDT use during pregnancy [29] but studies of RDT acceptability in other groups have been conducted. Such research in Ghana also highlighted mixed consequences of a negative test result [27, 28]: for example, some care providers of infants said that they would obtain an ACT elsewhere if none were prescribed [28]. Therefore, to maintain confidence in the RDTs – and particularly if their use is expanded significantly as the implementation of IST entails – it is important that health staff are aware of relevant local illness concepts and their relationship with biomedically defined malaria, and explain carefully the implications of the result.

The wider programme of research on the social and cultural context of MiP revealed that negative malaria test results also led to uncertainty among Ghanaian health staff [18]. Cases of pregnant women being treated with anti-malarials in spite of a negative result were identified. This resulted from a misinterpretation of the relevant policy document, which states that, in the absence of laboratory diagnosis, pregnant women with clinical symptoms of malaria should not be denied anti-malarials because the risk of not treating far outweighs the risks associated with over-treatment [30].

Over-treatment of malaria is however not restricted to pregnant women in Ghana. Research on malaria diagnoses across Africa has revealed a lack of confidence in tests and reliance on symptoms [31, 32]. For example, in northern Tanzania, over-treatment of malaria in general was linked to a range of factors, including health care staff members’ assumptions about malaria being the most important disease and patients’ expectations [31]. Although training can probably only go so far in terms of addressing the role of malaria treatment in terms of health staff satisfying the demands of their patients, were IST to be implemented, it is clear that any accompanying training should emphasize the importance of following case management guidelines.

A study in central Ghana on health staff’s knowledge of IPTp and case management during pregnancy revealed their varied awareness of the relevant guidelines [33]; almost 90% of health staff could describe all elements of the IPTp with SP policy, whereas 20 and 41% could correctly explain malaria case management policy in first or second/third trimesters, respectively. For these study respondents, having participated in a workshop on the IPTp policy strongly influenced whether they could correctly describe the policy. So, although the lack of knowledge about appropriate case management during pregnancy does not bode well for the implementation of IST, these results indicate that providing training can lead to high levels of awareness of policy guidelines.

For some pregnant women, the benefits of accurate diagnosis were also counterbalanced by the pain and discomfort of a finger prick. Indeed, trial staff were pessimistic about women’s readiness to undergo finger prick, but respondents were often willing to suffer the discomfort. Because of the additional benefits of participating in the clinical trial it is difficult to extrapolate this particular finding to the potential implementation of IST within ANC. However, from the broader study on the social and cultural context of MiP, it was clear that generally women trusted the advice of health staff and followed their instructions [18]. Furthermore, findings from the broader study, and research by Smith *et al*. [12] in central Ghana, emphasize how women tend to accept ANC as a package of services with relatively little attention to particular interventions. Given the often-hierarchical nature of interactions between pregnant women and health staff in ANC [21] it is also likely that many women would not refuse a finger prick for fear of reprimand or possible future refusal of care. Nonetheless, the uncertainty about the impact of finger pricks on ANC attendance highlights the need for further research linked to the implementation of IST as part of ‘normal’ ANC.

The reports of women demanding compensation for follow-up blood sampling are probably related to the context in which these data were collected and have little relevance for implementation within ANC; in such a clinical trial, there is often a tendency to incentivize any contribution to the study. For example, on clinic visits, participants are normally provided with a ‘travel allowance’ along with sodas and soap. Although small, such tokens set a precedent. They also signal a different balance of power in interactions with staff compared to that commonly encountered in ‘normal’ health care interactions; trial participants interpret their involvement as valuable and therefore make assumptions about compensation, whereas, in health facilities, help is sought and pregnant women (or any other group) are largely dependent on the heath staff (and often fear reprimand by refusing treatment).

### Anti-malarials for treatment or prevention

Interviewed trial participants complained about the side effects after taking SP for IPTp. This was also reflected in the wider study on the social and cultural context of MiP, which recorded complaints at every site (Central and Upper East Ghana, Kenya and Malawi) [18]. However, it is noteworthy that the complaints were most vociferous in the Upper East Ghana site. In spite of the complaints, across the sites reports of women not taking their SP tablets were rare, even – despite the policy of DOT – if they were given them to take at home or outside the consulting room. The cases of non-adherence were linked to personal experiences of vomiting after past doses. This was further indicative of women’s general desire to follow the instructions of health staff even though they experienced discomfort.

By contrast, the complaints about AL were indirect: trial staff reported women’s objections to the number of tablets that a treatment course entailed or the ‘strength’ of the anti-malarial. Nonetheless, trial staff were confident that the women completed the treatment courses despite these misgivings and, in this study, only one case of reported partial adherence to the AL trial regimen was reported by a trial participant. Nonetheless, given the use of a multiple-dose treatment regimen and, particularly during the possible roll-out, the absence of DOT for all but the first dose, the questions about the impact of such concerns on adherence are key for the success of IST.

Other research with non-pregnant participants has highlighted mixed levels of adherence to AL: 90% probably adherent in southwest Uganda [34], compared to 65% in southern Malawi [35] and 38% in northern Ethiopia [36]; whereas only 1.7% of over 500 respondents missed a dose in rural Tanzania [37]. The range of adherence results from the varied contexts in which the research was conducted and the study designs (including the definition of adherence) [36]. Informing participants that they would receive a follow-up visit has however been suggested to particularly improve adherence [36].

With regard to pregnant women, qualitative research at the same site in northern Ghana identified four respondents as having not completed their treatment course of anti-malarials during pregnancy; side effects played a role, as did confusion about the advice from health care staff, particularly if it contradicted ideas about malaria causation [18]. Meñaca *et al.* [16] also highlighted how, in Ghana, MiP-related health messages tended to focus on the use of non-prescribed and non-biomedical treatment during pregnancy, with little emphasis on adherence to prescribed anti-malarial regimens. To ensure the success of IST, related messages would have to emphasize the importance of following dosage instructions and completing a treatment course. Furthermore, appropriate health messaging is particularly important in endemic areas where MiP is often asymptomatic [1, 38] as is research on AL adherence among pregnant women.

## Conclusion

Despite the discomfort of the finger prick required to perform the intermittent malaria screening, trial participants generally expressed more positive sentiments towards IST-AL than IPTp-SP. This was largely a result of the range and severity of side effects directly associated with SP and the value that they placed on malaria (and broader disease) diagnosis. Nonetheless, questions remain about adherence to a multiple-dose anti-malarial treatment regimen during pregnancy, particularly in endemic areas where MiP is often non-symptomatic. Further research in a non-clinical trial context should address this issue. Any implementation of IST must be accompanied by appropriate health messages on adherence and the necessary training for health staff regarding case management guidelines.
